# The discrepancies between clinical and histopathological diagnoses of cardiomyopathies in patients with stage D heart failure undergoing heart transplantation

**DOI:** 10.1371/journal.pone.0269019

**Published:** 2022-06-01

**Authors:** Thana Lertsuttimetta, Monravee Tumkosit, Peerapat Kaveevorayan, Poonchavist Chantranuwatana, Nonthikorn Theerasuwipakorn, Pairoj Chattranukulchai, Sarinya Puwanant

**Affiliations:** 1 Division of Cardiovascular Medicine, Department of Medicine, Faculty of Medicine, Chulalongkorn University, Bangkok, Thailand; 2 Department of Radiology, Faculty of Medicine, Chulalongkorn University, Bangkok, Thailand; 3 Department of Pathology, Faculty of Medicine, Chulalongkorn University, Bangkok, Thailand; 4 Cardiac Center, King Chulalongkorn Memorial Hospital, Thai Red Cross Society, Bangkok, Thailand; University of Miami School of Medicine, UNITED STATES

## Abstract

**Background:**

This study aimed to determine the etiology of stage-D heart failure (HF) and the prevalence and prognosis of misdiagnosed cardiomyopathy in patients undergoing heart transplantation.

**Methods and results:**

We retrospectively reviewed 127 consecutive patients (mean age, 42 years; 90 [71%], male) from February 1994 to September 2021 admitted for heart transplant in our tertiary center. Pre-transplant clinical diagnosis was compared with post-transplant pathological diagnosis. The most common misdiagnosed cardiomyopathy was nonischemic cardiomyopathy accounting for 6% (n = 8) of all patients. Histopathological examination of explanted hearts in misdiagnosed patients revealed 2 arrhythmogenic cardiomyopathy, 2 sarcoidosis, 1 hypertrophic cardiomyopathy, 1 hypersensitivity myocarditis, 1 noncompacted cardiomyopathy, and 1 ischemic cardiomyopathy. Pre-transplant cardiac MRI and endomyocardial biopsy (EMB) were performed in 33 (26%) and 6 (5%) patients, respectively, with both performed in 3 (3% of patients). None of the patients undergoing both cardiac tests were misdiagnosed. During the 5-years follow-up period, 2 (25%) and 44 (37%) patients with and without pretransplant misdiagnosed cardiomyopathy died. There was no difference in survival rate between the groups (hazard ratio: 0.52; 95% CI:0.11–2.93; *P* = 0.314).

**Conclusions:**

The prevalence of misdiagnosed cardiomyopathy was 6% of patients with stage-D HF undergoing heart transplantation, the misdiagnosis mostly occurred in nonischemic/dilated cardiomyopathy. An accurate diagnosis of newly detected cardiomyopathy gives an opportunity for potentially reversing cardiomyopathy, including sarcoidosis or myocarditis. This strategy may minimize the need for advanced HF therapy or heart transplantation. With advances in cardiac imaging, improvements in diagnostic accuracy of the etiology of HF can improve targeting of treatment.

## Introduction

Heart failure is a common clinical syndrome caused by various cardiovascular diseases [1–3]. Evaluation of heart failure etiology is crucial and should be correctly and rapidly identified. An accurate diagnosis provides an opportunity for specific treatment to be delivered that can potentially reverse heart failure and improve overall prognosis. In inherited cardiomyopathy, an accurate diagnosis also helps in prevention and screening all affected family members. Histopathological analysis of the explanted heart can reveal the definite diagnosis of the etiology of heart failure in patients undergoing heart transplantation. The linkages between histopathological examination, patient genotype-phenotype, clinical diagnosis, and findings of cardiac imaging and cardiac investigations is a pivotal pathway to better understand pathophysiology, nature of diseases, caveats in clinical diagnosis, and phenotypic manifestation of the etiologies of heart failure and cardiomyopathies. Previous studies have shown a discrepancy rate between clinical diagnosis and pathological diagnosis ranging between 7% to 23% of explanted heart examinations [4–11]. However, most of the studies were investigated in the years when the performance rate of cardiac magnetic resonance imaging (MRI) was low [4, 5, 9]. Most of these studies were also examined in non-Asian patient populations [4–8, 10]. This study aimed to determine: (1) the etiology of end-stage heart failure, (2) the prevalence of misdiagnosis of cardiomyopathy type and the discrepancy rate between pre-transplant /clinical diagnosis and post-transplant/ histopathological diagnosis, (3) the performance of pre-transplant cardiac investigations in patients with misdiagnoses of the etiology of heart failure, and (4) the post-transplant survival rate of patients with misdiagnosis.

## Methods

### Study population

The study protocol was approved by the Chulalongkorn University’s Institutional Review Board with a waiver of written informed consent (IRB 796/63). All methods were carried out in accordance with the tenets of the Declaration of Helsinki and the ethical standard guidelines and regulations. We retrospectively reviewed histopathological findings and pre-transplant clinical diagnoses of explanted hearts in consecutive patients with stage D heart failure undergoing heart transplantation in our tertiary center from February 1994 to September 2021. Stage D heart failure was defined as advanced heart failure with one of the following clinical features indicating high mortality: persistent NYHA class III-IV, elevated natriuretic peptide, inotrope dependence, frequent heart failure hospitalizations, end-organ dysfunction, frequent defibrillator shocks, high-dose diuretic requirement, hyponatremia, hypotension, cachexia, intolerance to guideline-directed medical therapy, or low peak oxygen consumption [12–15]. Patients with unavailable or incomplete histopathological specimens for analysis were excluded. Patient demographics and clinical, laboratory, and histopathological data were extracted from medical records. Echocardiography, coronary angiography, cardiac MRI, and nuclear scan were reviewed from the cardiovascular and radiology picture archiving and communication (PAC) system. Pre-transplant/clinical diagnosis was determined by the heart failure and transplant cardiologists using clinical, echocardiographic, cardiac MRI, coronary angiogram, and cardiac endomyocardial (EMB) information before heart transplantation. Post-transplant/histopathological diagnosis was determined by cardiac pathologists on explanted hearts in a routine clinical examination. The etiologies of heart failure were classified as ischemic cardiomyopathy (ISCM) and nonischemic cardiomyopathy (NISCM). ISCM was defined as the presence of ≥ 70% luminal stenosis in the major epicardial coronary branches, or ≥ 50% luminal stenosis in the left main coronary artery assessed by gross pathological analysis or on coronary angiogram with myocardial scar or replacement fibrosis [16, 17]. NISCM was subclassified into one of the following types: dilated and/or familial NISCM, valvular cardiomyopathy, hypertrophic cardiomyopathy (HCM), peripartum cardiomyopathy, noncompacted cardiomyopathy, alcoholic cardiomyopathy, arrhythmogenic right ventricular cardiomyopathy (ARVC) and/or left dominant arrhythmogenic cardiomyopathy (LDAC), cardiac amyloidosis, myocarditis, cardiac sarcoidosis, congenital heart disease, and cardiac tumor.

### Cardiac investigations

Transthoracic echocardiographic studies were performed in all patients using commercially available ultrasound machines, Epiq CVX and IE33 (Philips Medical Systems, Andover, MA), Vivid 7 (GE Healthcare, Vingmed Ultrasound, Horten Norway), and Prosound Alpha 10 (Hitachi Aloka Medical. Ltd., Tokyo, Japan). Clinically indicated cardiac MRIs were performed on 1.5 -T scanners (Signa HD, GE Healthcare, Milwaukee, USA; MAGNETOM Aera, Siemens Healthineers, Germany) and 3.0-T scanners (Achieva, Phillips Healthcare, Best, The Netherlands; MAGNETOM Skyra; MAGNETOM Vida, Siemens Healthineers, Germany) using electrocardiographic and respiratory gating. Steady state free processing cine images, first-pass myocardial perfusion and late gadolinium-enhancement images were acquired as per standardized protocols [18]. Coronary angiography and endomyocardial biopsy with ≥ 4 sampling tissues were performed using standard techniques [19, 20].

### Pathological analysis

Histological examinations of pre-transplant endomyocardial sampling tissues were performed in patients undergoing pre-transplant EMB. Gross and histological explanted hearts were examined in all study patients. The specimens were fixed in formalin. A histological 5-micron thick section sampling from the left and right ventricles, interventricular septum, valves, and coronary vessels were stained for hematoxylin and eosin and Masson’s trichrome. In patients with clinically suspicious amyloid cardiomyopathy and myocarditis, Congo red stain and immunohistochemical analysis were performed.

### Statistical analysis

Categorical and continuous data were expressed as frequency (percentage) and mean ± SD, respectively. Differences between variables were compared by a student’s t-test for continuous variables with normal distribution and a Wilcoxon-rank sum test for continuous variables with non-normal distribution. Categorical variables were compared using Chi’s square test or Fisher exact test, where appropriate. Kaplan-Meier curves were constructed to estimate survival among patients with concordant and discordant diagnosis between pre-transplant clinical and post-transplant pathological diagnosis. The data were analyzed using software IBM^®^ SPSS^®^ statistics version 28.0.0.0 and JMP statistical discovery software. P < 0.05 was considered statistically significant.

## Results

From February 1994 to September 2021, 146 patients underwent cardiac transplantation. Of these, 19 patients were excluded because their pathological specimens were not available for analysis. A total of 127 patients were included in the study (mean age, 42 years; 90 [71%], male). All these patients met the criteria of stage D heart failure. Table 1 illustrates baseline patient characteristics. Pre-transplant intravenous inotrope or cardiogenic shock (INTERMACS profile 1–3) were present in 29% of patients. Mechanical circulatory support devices were implanted in 7 patients (6%). The most common (76%) pre-transplant/clinical diagnosis was NISCM.

**Table 1:** Baseline patient characteristics.

	Overall (n = 127)
Age (years)	42 ± 15
Male (n, %)	90 (71)
BMI (kg/m^2^)	21± 4
Pre-CIED (n, %) *	64 (50)
INTERMACS 1–3	37 (29)
IABP at heart transplant	7 (6)
MCS at heart transplant	7 (6)
Ischemic time (min)	229 ± 63
History of CABG (n, %)	6 (5)
History of PCI (n, %)	15 (12)
Co-morbidities (n, %)	
Hypertension	15 (12)
Diabetes mellitus	11 (9)
Dyslipidemia	22 (17)
Ischemic stroke	15 (12)
Tobacco use (n, %)	37 (29)
Family history of cardiomyopathy/sudden cardiac death (n, %)	26 (21)
History of anticoagulation prior to transplant (n, %)	68 (54)
Pre transplant clinical diagnosis (n, %)	
Ischemic cardiomyopathy	30 (24)
Nonischemic cardiomyopathy	97 (76)
Idiopathic/familial non-ischemic cardiomyopathy	68 (54)
Valvular cardiomyopathy	7 (6)
Hypertrophic cardiomyopathy	6 (5)
Congenital heart disease	6 (5)
ARVC or LDAC	5 (4)
Peripartum cardiomyopathy	2 (2)
Cardiac amyloidosis	1 (1)
Myocarditis	1 (1)
Cardiac myxoma	1 (1)
Non-compacted cardiomyopathy	0
Cardiac sarcoidosis	0
Cardiac Investigation (n, %)	
Echocardiography	127 (100)
Coronary angiography	93 (73)
Cardiac MRI	33 (26)
Cardiac EMB	6 (5)
Cardiac MRI and EMB	3 (2)
Nuclear scan	2 (2)

ARVC: Arrhythmogenic right ventricular cardiomyopathy; BMI: Body Mass Index; CABG: Coronary artery bypass graft; CIED: Cardiac implantable electronic device; EMB: Endomyocardial biopsy; IABP: Intraaortic balloon pump; LDAC: left dominant arrhythmogenic cardiomyopathy; MCS: Mechanical Circulatory Support; MRI: Magnetic Resonance Imaging; PCI: Percutaneous coronary intervention

### Misdiagnosis of cardiomyopathy

Of 127 patients, 8 (6%) patients were clinically misdiagnosed, and 5 (4%) patients had additional findings on pathological analysis (Table 2). All 8 patients (100%) with a pre-transplant misdiagnosis of cardiomyopathy were clinically diagnosed as NISCM/myocarditis pretransplant. Histopathological examination of these explanted hearts revealed 2 ARVC/LDAC, 2 cardiac sarcoidosis, 1 end-stage HCM, 1 hypersensitivity myocarditis, 1 noncompacted cardiomyopathy, and 1 ischemic cardiomyopathy. One patient with a pre-transplant diagnosis of ARVC was found to have cardiac sarcoidosis on pathological examination of the explanted heart. One patient with valvular cardiomyopathy who developed multiple episodes of prosthetic paravalvular leak was found to have Takayasu’s arteritis of the aorta in addition to valvular (nonischemic) cardiomyopathy on pathological examination. Those patients with pathologically proven diagnosis of reversible cardiomyopathies or aortic disorder including cardiac sarcoidosis (n = 2), Takayasu’s arteritis (n = 1), or hypersensitivity myocarditis (n = 1) were not treated with corticosteroid or immunosuppressive agents prior to heart transplantation.

**Table 2:** Histopathological diagnoses of the entire cohort.

Histopathological (final) diagnosis	n	Concordant	Discordant		Additional Findings
Total	**127**	**119 (94%)**	**8 (6%)**		**5 (4%)**
Ischemic cardiomyopathy	31 (24.4%)	30 (97%)	1 (3% [1/31])	Misdiagnosed as pretransplant non-ISCM (n = 1)	
Idiopathic/familial non-ischemic cardiomyopathy	58 (46.6%)	58 (100%)	0		Concomitant CAD (n = 2) Concomitant anomalous coronary artery (n = 1), moderate area of myocarditis (n = 1)
Hypertrophic cardiomyopathy	8 (6.3%)	6 (75%)	1 (13% [1/8])	Misdiagnosed as pretransplant non-ISCM (n = 1)	
ARVC and LDAC	6 (4.7%)	4 (67%)	2 (25% [2/6])	Misdiagnosed as pretransplant non-ISCM (n = 2)	
Non-compacted cardiomyopathy	1 (0.8%)	1 (50%)	1 (100% [1/1])	Misdiagnosed as pretransplant myocarditis (n = 1)	
Peripartum cardiomyopathy	2 (1.6%)	2 (100%)	0		
Cardiac sarcoidosis	2 (1.6%)	0 (0%)	2 (100% [2/2])	Misdiagnosed as pretransplant non-ISCM (n = 1) and ARVD (n = 1)	
Valvular cardiomyopathy	8 (6.3%)	8 (100%)	0		Takayasu’s arteritis (n = 1)
Congenital heart disease	6 (4.7%)	6 (100%)	0		
Hypersensitivity myocarditis	1 (0.8%)	0	1 (100% [1/1])	Misdiagnosed as pretransplant non-ISCM (n = 1)	
Myocarditis	2 (1.6%)	2 (100%)	0		
Cardiac amyloidosis	1 (0.8%)	1 (100%)	0		
Cardiac myxoma	1 (0.8%)	1 (100%)	0		

ARVC: Arrhythmogenic right ventricular cardiomyopathy; CAD: coronary artery disease; LDAC: left dominant arrhythmogenic cardiomyopathy; Non-ISCM: Non- ischemic cardiomyopathy

### Pre-transplant multi-modality imaging and EMB

**Fig 1:**
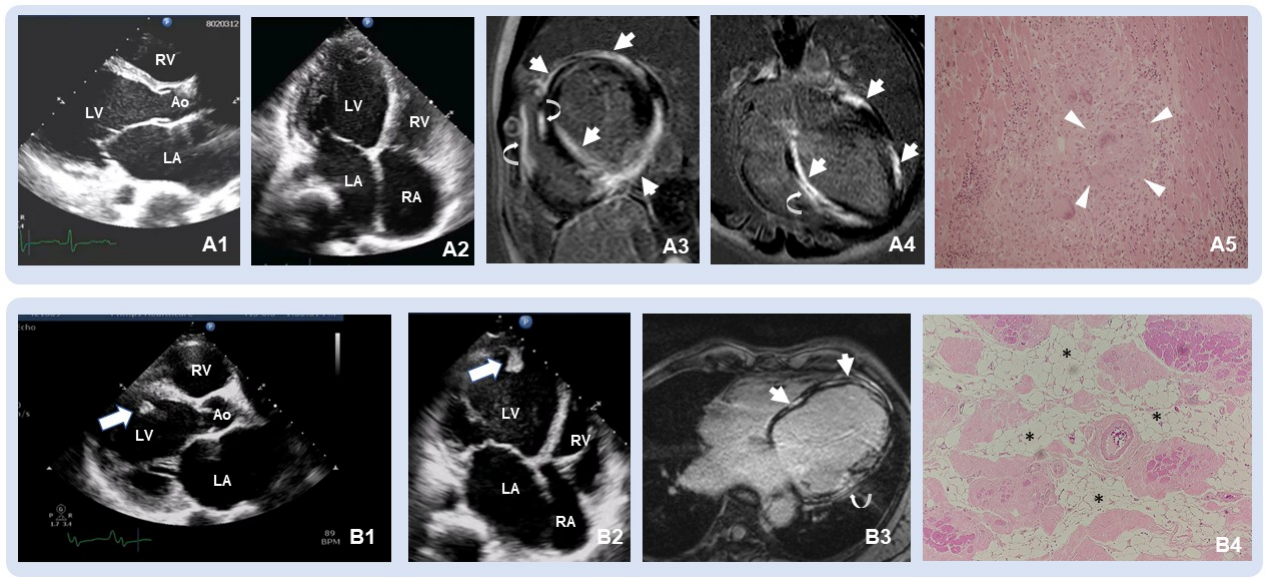
Pre-transplant multimodality imaging and post-transplant histopathological findings from two patients who were misdiagnosed. Multi-imaging modalities and histopathological findings of patient #2 (Table 3) with a post-transplant diagnosis of cardiac sarcoidosis and patient #5 (Table 3) with a post-transplant diagnosis of left dominant arrhythmogenic cardiomyopathy (LDAC). **A1-2**, a transthoracic echocardiogram showing dilated left ventricle (LV) with reduced LV wall thickness and severe LV systolic dysfunction. **A3-4**, cardiac MRI images showing extensive subepicardial late gadolinium enhancement of anterior and lateral walls and interventricular septum (arrows) and transmural late gadolinium enhancement of inferior wall (arrows) and late gadolinium enhancement of right ventricular free wall and septum (curved arrows). **A5**, histological micrograph (x20) from an explanted heart showing noncaseating, multinucleated giant cell granuloma (arrow heads) involved left ventricular myocardium consistent with cardiac sarcoidosis. **B1-2**, a transthoracic echocardiogram showing severe LV systolic dysfunction with apical thrombus (white arrows). **B3**, a 4-chamber-view cardiac MRI image showing mid-wall late gadolinium enhancement of interventricular septum and LV apex (arrows) and severe myocardial thinning with late gadolinium enhancement of lateral wall (curved arrows). **B4**, left ventricular histological micrograph showing fibroadipose replacement (asterisks) of the compacted myocardium consistent with arrhythmogenic cardiomyopathy.

A pre-transplant echocardiogram was performed in all patients. Pre-transplant cardiac MRI, EMB, and both MRI and EMB were performed in 33 (26%), 6 (5%), 3 (2%) patients, respectively. ICDs were implanted in 50% of patients at the time of clinical evaluation. Of 6 patients undergoing pre-transplant EMBs, 3 were to rule out myocarditis (1 confirmed myocarditis), 1 was to rule out cardiac sarcoidosis in a patient with clinical diagnoses of ARVC (1 revealed nonspecific findings), 1 was to confirm cardiac amyloidosis (1 were negative for cardiac amyloidosis despite 8 sampling tissues), and 1 was to find the cause of restrictive cardiomyopathy (1 confirmed HCM). Table 3 shows pre-transplant performances of multi-modality imaging and EMB in 8 patients with misdiagnosed etiologies of heart failure. Of these, EMB was performed in only one patient (13%) and cardiac MRIs were not performed in 50% of patients (4 of 8) due to ICD implantation (n = 3) and extracorporeal membrane oxygenator (ECMO) implantation with cardiogenic shock (n = 1). Pre-transplant echocardiography in these 8 patients who were misdiagnosed pretransplant presented identical features of dilated cardiomyopathy, generalized left ventricular (LV) systolic dysfunction with normal and/or reduced LV wall thickness, without clues of specific cardiomyopathies. Fig 1(A1-A5) and S1 Video illustrate pre-transplant echocardiogram and cardiac MRI in a patient 5 with pre-transplant NISCM and post-transplant pathological findings of cardiac sarcoidosis. Fig 1(B1-B4) and S2 Video illustrate pre-transplant echocardiogram and cardiac MRI in a patient 2 with pre-transplant NISCM and post-transplant pathological findings of LDAC. The rate of misdiagnosis in patients undergoing cardiac MRI or EMB pretransplant was similar to those who did not undergo cardiac MRI or EMB pretransplant (9% [3 of 33] vs. 5% [5 of 94], p = 0.442 for cardiac MRI; and 17% [1 of 6] vs. 8% [7 of 121], p = 0284 for EMB). None of the patients undergoing cardiac MRI and EMB (n = 3) were misdiagnosed. Among patients with pathologically proven NISCM (n = 96), the rates of error in diagnosis among patients with and without pre-transplant cardiac MRI and EMB were consistent with those of the entire cohort. Table 4 shows ancillary pathological findings in addition to pre-transplant correctly identified main type of cardiomyopathy: concomitant significant single-vessel coronary artery diseases found in 2 (2%), anomalous coronary artery in 1 (1%), and histological evidence of small area of previous myocarditis in 1 (1%) of 96 patients with pathologically proven NISCM.

**Table 3:** Multimodality imaging in 8 patients with discordant diagnoses.

	Post-transplant / histopathological diagnosis	Pre transplant/clinical diagnosis	Pre-transplant echocardiogram	Pre-transplant coronary angiogram	Pre-transplant cardiac MRI	Pre-transplant EMB
Patient #1	HCM	NISCM	Yes	Yes	**No**	**No**
Patient #2	LDAC	NISCM	Yes	Yes	**No**	**No**
Patient #3	ARVC	NISCM	Yes	Yes	Yes	**No**
Patient #4	Cardiac sarcoidosis	ARVC	Yes	**No**	Yes	**No**
Patient #5	Cardiac sarcoidosis	NISCM	Yes	Yes	Yes	**No**
Patient #6	ISCM	NISCM	Yes	Yes	**No**	**No**
Patient #7	LVNC	Myocarditis with cardiogenic shock, on ECMO	Yes	Yes	**No**	Yes
Patient #8	Hypersensitivity myocarditis	Alcoholic CM	Yes	Yes	**No**	**No**

ARVC: Arrhythmogenic right ventricular cardiomyopathy; CM: Cardiomyopathy, ECMO: extracorporeal membrane oxygenator; EMB: Endomyocardial biopsy; HCM: Hypertrophic cardiomyopathy; LDAC: left dominant arrhythmogenic cardiomyopathy; ISCM: Ischemic cardiomyopathy; LVNC: Left ventricular non-compaction cardiomyopathy; MRI: Magnetic Resonance Imaging; NISCM: non-ischemic cardiomyopathy.

**Table 4:** Multimodality imaging in 5 patients with additional findings.

	Main Histopathological diagnosis	Additional findings	Pre-transplant echocardiography	Pre-transplant coronary angiogram	Pre-transplant cardiac MRI	Pre-transplant EMB
Patient A	NISCM	Coronary artery disease (50% LAD stenosis and old myocardial infarction scar in posterior wall)	Yes	Yes	No	No
Patient B	NISCM	Non-active myocarditis (small area)	Yes	Yes	No	No
Patient C	NISCM	Anomalous coronary artery	Yes	No	Yes	No
Patient D	NISCM	Coronary artery disease (50% stenosis of left main coronary artery)	Yes	Yes	No	No
Patient E	NISCM	Takayasu aortitis of the aorta	Yes	Yes	No	No

CM: Cardiomyopathy; EMB: Endomyocardial biopsy; NISCM: non-ischemic cardiomyopathy.

### Post-transplant prognosis of misdiagnosed cardiomyopathy

**Fig 2:**
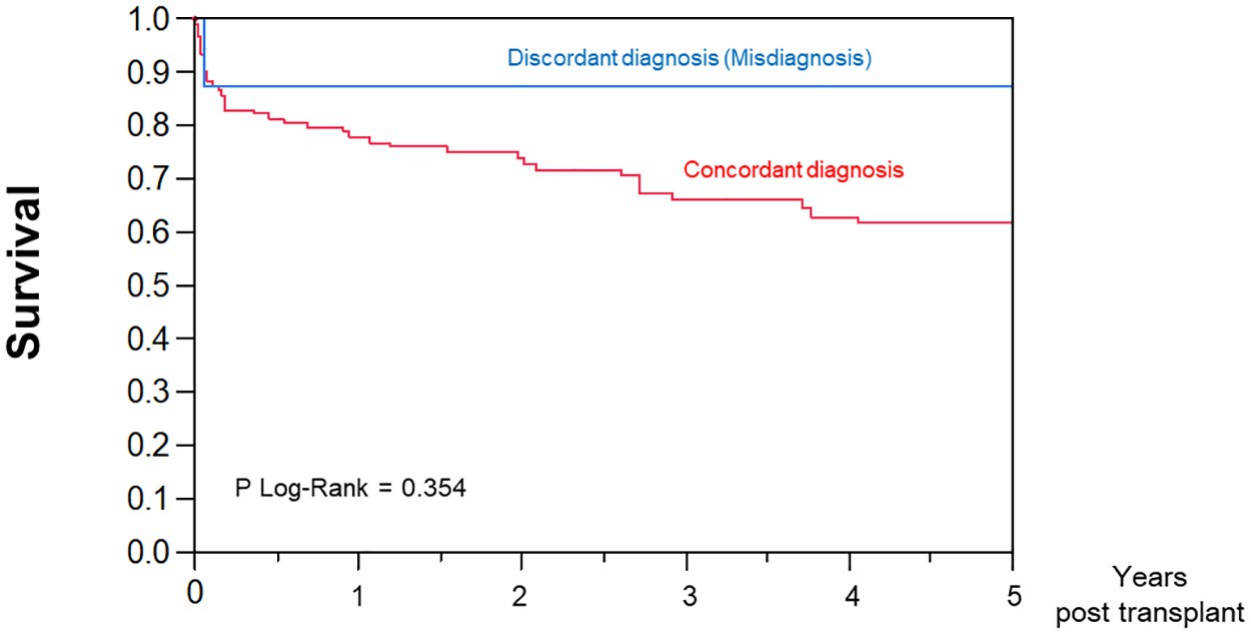
Survival curves among patients with concordant and discordant diagnosis between pre-transplant clinical diagnosis and post-transplant pathological findings.

During the 5-years follow-up period, 2 (25%) and 44 (37%) patients with and without pretransplant misdiagnosed cardiomyopathy died. There was no difference in survival rate between the groups (hazard ratio: 0.52; 95% CI:0.11–2.93; *P* = 0.314) (Fig 2).

## Discussion

The major findings of our study are: (1) misdiagnosis of the etiology of end-stage heart failure in patients undergoing heart transplantation in our study were found in 6% of patients, (2) the most common etiology of stage D or end-stage heart failure in patients undergoing heart transplantation is NISCM, (3) the misdiagnosis of cardiomyopathy mostly occurred with a clinical/ pre-transplant diagnosis of NISCM, (4) the most common reversible cardiomyopathy with pre-transplant misdiagnoses were cardiac sarcoidosis and hypersensitivity myocarditis, all of these patients did not undergo EMB pre-transplant or received corticosteroid or immunosuppressive agents, (5) pre-transplant EMBs with 8 sampling tissues were found to be negative for amyloid fibril by Congo-red staining and polarized examination in a patient with a clinical diagnosis of transthyretin cardiac amyloidosis, (6) 3% of patients (2 out of 58) with NISCM had additional concomitant significant coronary artery diseases not identified pretransplant, (7) the misdiagnosis rate was not different between patients who underwent both EMB and cardiac MRI and those who did not undergo both cardiac investigations, and (8) survival post-transplant between accurate diagnosis and misdiagnosis were similar. These findings represent the first reported data in Southeast Asian patient population.

The errors or misdiagnoses of the etiology of end-stage heart failure in patients undergoing heart transplantation in our study were found in 6% of patients. This error rate found in our study was similar to a recent study by Lin et al. [7] that reported 7% of 338 patients undergoing heart transplantation from 2004 through 2017 having errors in identifying the etiologies of heart failure. A total of 18% and 10% of their patients underwent cardiac MRI and EMB pretransplant, respectively [7]. In our study, the rate of cardiac MRI (27%) was higher, while the rate of EMB was lower (5%). Angelini et al. reported that 8% of 257 patients who underwent heart transplantation during 1985–1994 were misdiagnosed [4]. Interestingly, the rate of misdiagnosis was only 8% when cardiac MRI was not widely available. This may be due to high rate of EMB in their study, covering 25% of study patients. The discrepancy rate between pre-transplant clinical diagnosis and post-transplant pathological diagnosis were prevalent in Luk’s and Roberts’ studies [5, 6]. Luk et al. identified 17% of patients undergoing heart transplantation from 1987 to 2006 being misdiagnosed, while Robert et al. found that 13% of 314 patients undergoing heart transplantation from 1993 to 2012 had misdiagnosed etiologies [5, 6]. One of the main possible reasons of the higher misdiagnoses rates in both studies may be associated with the lack of access to cardiac MRI and low EMB rate during those study years. In our study, the rate of misdiagnosis in patients undergoing both cardiac MRI and EMB were similar to those without undergoing both investigations. These findings were in contrast to the study by Lin et al [7]. They found that performance of cardiac MRI was significantly associated with fewer errors in pre-transplant/clinical diagnosis in patients with NISCM [7]. The possible explanation of the difference between the studies remained unclear. We hypothesized that the misdiagnosed cardiomyopathy in our study (cardiac sarcoidosis, ARVC/ LDAC, or burned out HCM) were difficult to accurately diagnose and subject to error despite cardiac MRI/EMB. These may be associated with a lack of sensitivity of the clinical diagnostic criteria in those cardiomyopathies and scattered disease pattern on myocardial involvement resulting in a false negative in diagnosis [21–29]. In a recent study by Perazzolo et al., not all patients who underwent EMB and cardiac MRI were diagnosed as ARVC/LDAC or cardiac sarcoidosis [29]. EMB was diagnostic for ARVC and cardiac sarcoidosis in 69% and 13–65% of patients, [25–27, 29–31] respectively. In our study, the frequent errors in diagnosis included cardiac sarcoidosis, hypersensitivity myocarditis, non-compacted cardiomyopathy, and ARVC/LDAC. Similarly, Roberts et al. found that the most prevalent misdiagnoses were ARVC (100%), cardiac sarcoidosis (100%), noncompacted cardiomyopathy (100%), and HCM (41%) [6]. Lin et al. demonstrated that the most frequently misdiagnosed rates among NISCM were found in pathologically proven ARVC/LDAC (38%), cardiac sarcoidosis (33%), and HCM (14%) [7].

We found one patient (1% of study cohort) with a clinical diagnosis of alcoholic cardiomyopathy with dobutamine dependence who turned out to have a pathological diagnosis of hypersensitivity myocarditis. The prevalence of hypersensitivity myocarditis in our study was lower than the 2.7% rate found in a large study by Yoshizawa et al. [32]. About half of these patients had received dobutamine infusion. Our findings underscore how an accurate diagnosis of reversible cardiomyopathy can identify specific treatments including corticosteroid and immunosuppressive agents.

We also demonstrated that 1% of our patients who were diagnosed as NISCM pretransplant were found to have ISCM on histopathological examination. In contrast, 3% of patients with pathologically proven NISCM were found to have concomitant significant coronary artery diseases on pathological analysis. Two of these patients were not identified pretransplant despite undergoing pretransplant coronary angiography. Notably, 73% of our cohort underwent pre-transplant coronary angiography. The angiography rate in our study was similar to the 79% rate in the Lin’s study [7]. They found that 12% of patients who were diagnosed as NISCM pretransplant was found to have ISCM on pathological analysis. The higher rate of errors in identifying the NISCM in their study may be related to the higher prevalence of post-transplant ISCM (47%) compared to our study (24%). Mehra et al recently investigated 112 patients with the diagnosis of NISCM at the time of heart transplant [10]. They demonstrated that 21% of their cohort were pathologically reclassified as ISCM defined as severe 3-vessel disease with myocardial scar. Subsequently, 33% of them were reclassified as > 1 vessel moderate to severe coronary artery diseases without myocardial scar. However, they did not describe the proportion or numbers of patients undergoing pretransplant coronary angiogram or noninvasive testing.

Recently, there has been an increasing recognition that nonobstructive coronary artery disease can be present in patients with myocardial infarction and ISCM evidence assessed by late gadolinium enhancement [7, 33–35]. Although coronary angiography is the gold standard to determine anatomical obstructive coronary artery disease, spontaneous recanalization of coronary lumen and non-epicardial coronary diseases can occur in some patients [7, 36].

In the present analysis, we found one patient with pathologically proven cardiac amyloidosis who had undergone EMBs (2 episodes of EMBs consisting of 8 samples) in the setting of highly suspicious cardiac amyloidosis that revealed no amyloid fibril on pretransplant histopathological analysis. However, pre-transplant cardiac MRI and echocardiogram were strongly consistent with infiltrative cardiomyopathy. Serum free light chain and serum and urine immunofixation were all negative. This patient did not undergo a PYP scan before transplantation. These findings highlight that negative endocardial biopsy in pathologically proven cardiac amyloidosis is rare but can occur. Pellika et al. studied the yield of endomyocardial biopsy in 30 patients with cardiac amyloidosis and found that amyloid was present in only one of four specimens in 2 of 30 patients undergoing EMB suggesting the possibility of sampling error [37]. Ananthakrishna et al. reported a case with light chain cardiac amyloidosis with an initial negative EMB and a subsequently confirmed cardiac amyloidosis [38].

### Study limitations

Our findings are limited to patients with stage D heart failure undergoing heart transplantation and may not be generalizable to patients with stage C heart failure and stage D heart failure who were treated with other therapies. A new technique of cardiac MRI was incorporated during the last few years of study accounting for 15% of the entire cohort. This improved technique likely impacted diagnostic accuracy. Interobserver variabilities among the experts in echocardiographic, coronary angiographic, cardiac MRI, and histopathological interpretation were not performed in the present study.

## Conclusions

Among patients with stage D heart failure undergoing heart transplantation, the most common etiology of heart failure is NISCM. A discrepancy between pre-transplant clinical diagnosis and histopathological diagnosis by explanted heart analysis occurred in 6% of our study patients. Cardiac sarcoidosis and hypersensitivity myocarditis were the most common misdiagnoses followed by noncompacted cardiomyopathy and ARVC/LDAC. Those patients with a pre-transplant misdiagnosis clinically presented as chronic worsening heart failure with typical echocardiographic feature of idiopathic dilated/nonischemic cardiomyopathy, dilated and generalized LV systolic dysfunction with a normal or reduced LV wall thickness, and no echocardiographic clue of specific cardiomyopathies. Our findings underscore that an accurate diagnosis of a newly diagnosed cardiomyopathy, especially non-ischemic type, give an opportunity for specific treatment that can potentially reverse of cardiomyopathy and heart failure. This strategy may minimize the need for advanced heart failure therapy or heart transplantation. With the advent of new techniques in modern echocardiography, cardiac MRI, nuclear cardiology, and coronary artery imaging, improvements in diagnostic accuracy of the etiology of heart failure should follow.

## Supporting information

S1 Table Baseline patient characteristics. (DOCX)

S2 Table Histopathological diagnoses of the entire cohort. (DOCX)

S3 Table A. Multimodality imaging in 8 patients with discordant diagnoses. B. Multimodality imaging in 5 patients with additional findings.
(DOCX)

S1 Fig Pre-transplant multimodality imaging and post-transplant histopathological findings from two patients who were misdiagnosed. (TIF)

S2 Fig Survival curves among patients with concordant and discordant diagnosis between pre-transplant clinical diagnosis and post-transplant pathological findings. (TIF)

S1 Video Pre-transplant echocardiogram in a patient with pre-transplant nonischemic cardiomyopathy and post-transplant pathological findings of cardiac sarcoidosis. (MP4)

S2 Video Pre-transplant echocardiogram in a patient with pre-transplant nonischemic cardiomyopathy and post-transplant pathological findings of LDAC. (MP4)

S1 File Minimal data set. (DOCX)
