# Supplementary material for: The discrepancies between clinical and histopathological diagnoses of cardiomyopathies in patients with stage D heart failure undergoing heart transplantation
Source: PLoS One. 2022 Jun 1;17(6):e0269019. doi: 10.1371/journal.pone.0269019 (PMC9159581; doi:10.1371/journal.pone.0269019)
Supplement: S1 Table — (DOCX) [file pone.0269019.s001.docx]

**S1 Table 1 Baseline patient characteristics**

|  | **Overall**  **(n=127)** |
| --- | --- |
| Age (years) | 42 ± 15 |
| Male (n, %) | 90 (71) |
| BMI (kg/m^2^) | 21± 4 |
| Pre-CIED (n, %) * | 64 (50) |
| INTERMACS 1-3 | 37 (29) |
| IABP at heart transplant | 7 (6) |
| MCS at heart transplant | 7 (6) |
| Ischemic time (min) | 229 ± 63 |
| History of CABG (n, %) | 6 (5) |
| History of PCI (n, %) | 15 (12) |
| Co-morbidities (n, %) |  |
| Hypertension | 15 (12) |
| Diabetes mellitus | 11 (9) |
| Dyslipidemia | 22 (17) |
| Ischemic stroke | 15 (12) |
| Tobacco use (n, %) | 37 (29) |
| Family history of cardiomyopathy/sudden cardiac death (n, %) | 26 (21) |
| History of anticoagulation prior to transplant (n, %) | 68 (54) |
| Pre transplant clinical diagnosis (n, %) |  |
| Ischemic cardiomyopathy | 30 (24) |
| Nonischemic cardiomyopathy | 97 (76) |
| Idiopathic/familial non-ischemic cardiomyopathy | 68 (54) |
| Valvular cardiomyopathy | 7 (6) |
| Hypertrophic cardiomyopathy | 6 (5) |
| Congenital heart disease | 6 (5) |
| ARVC or LDAC | 5 (4) |
| Peripartum cardiomyopathy | 2 (2) |
| Cardiac amyloidosis | 1 (1) |
| Myocarditis | 1 (1) |
| Cardiac myxoma | 1 (1) |
| Non-compacted cardiomyopathy | 0 |
| Cardiac sarcoidosis | 0 |
| Cardiac Investigation (n, %) |  |
| Echocardiography | 127 (100) |
| Coronary angiography | 93 (73) |
| Cardiac MRI | 33 (26) |
| Cardiac EMB | 6 (5) |
| Cardiac MRI and EMB | 3 (2) |
| Nuclear scan | 2 (2) |

ARVC: Arrhythmogenic right ventricular cardiomyopathy; BMI: Body Mass Index; CABG: Coronary artery bypass graft; CIED: Cardiac implantable electronic device; EMB: Endomyocardial biopsy; IABP: Intraaortic balloon pump; LDAC: left dominant arrhythmogenic cardiomyopathy; MCS: Mechanical Circulatory Support; MRI: Magnetic Resonance Imaging; PCI: Percutaneous coronary intervention
