# Supplementary material for: The discrepancies between clinical and histopathological diagnoses of cardiomyopathies in patients with stage D heart failure undergoing heart transplantation
Source: PLoS One. 2022 Jun 1;17(6):e0269019. doi: 10.1371/journal.pone.0269019 (PMC9159581; doi:10.1371/journal.pone.0269019)
Supplement: S2 Table — (DOCX) [file pone.0269019.s002.docx]

**S2 Table 2 Histopathological diagnoses of the entire cohort**

| **Histopathological (final) diagnosis** | **n** | **Concordant** | **Discordant** | | | **Additional Findings** |
| --- | --- | --- | --- | --- | --- | --- |
| Total | **127** | **119 (94%)** | **8 (6%)** | | | **5 (4%)** |
| Ischemic cardiomyopathy | 31  (24.4 %) | 30  (97%) | 1  (3%  [1/31]) | Misdiagnosed as pretransplant non-ISCM (n=1) |  | |
| Idiopathic/familial non-ischemic cardiomyopathy | 58  (46.6 %) | 58  (100%) | 0 |  | Concomitant CAD (n=2)  Concomitant anomalous coronary artery (n=1), moderate area of myocarditis (n=1) | |
| Hypertrophic cardiomyopathy | 8  (6.3 %) | 6  (75%) | 1  (13%  [1/8]) | Misdiagnosed as pretransplant non-ISCM (n=1) |  | |
| ARVC and LDAC | 6  (4.7 %) | 4  (67%) | 2  (25%  [2/6]) | Misdiagnosed as pretransplant non-ISCM (n=2) |  | |
| Non-compacted cardiomyopathy | 1  (0.8 %) | 1  (50%) | 1  (100%  [1/1]) | Misdiagnosed as pretransplant myocarditis (n=1) |  | |
| Peripartum cardiomyopathy | 2  (1.6 %) | 2  (100%) | 0 |  |  | |
| Cardiac sarcoidosis | 2  (1.6 %) | 0 (0%) | 2  (100% [2/2]) | Misdiagnosed as  pretransplant non-ISCM (n=1) and ARVD (n=1) |  | |
| Valvular cardiomyopathy | 8  (6.3 %) | 8  (100%) | 0 |  | Takayasu’s arteritis (n=1) | |
| Congenital heart disease | 6  (4.7 %) | 6  (100%) | 0 |  |  | |
| Hypersensitivity myocarditis | 1  (0.8 %) | 0 | 1  (100%  [1/1]) | Misdiagnosed as pretransplant non-ISCM (n=1) |  | |
| Myocarditis | 2  (1.6 %) | 2  (100%) | 0 |  |  | |
| Cardiac amyloidosis | 1  (0.8 %) | 1  (100%) | 0 |  |  | |
| Cardiac myxoma | 1  (0.8 %) | 1  (100%) | 0 |  |  | |

ARVC: Arrhythmogenic right ventricular cardiomyopathy; CAD: coronary artery disease; LDAC: left dominant arrhythmogenic cardiomyopathy; Non-ISCM: Non- ischemic cardiomyopathy
