# Supplementary material for: The discrepancies between clinical and histopathological diagnoses of cardiomyopathies in patients with stage D heart failure undergoing heart transplantation
Source: PLoS One. 2022 Jun 1;17(6):e0269019. doi: 10.1371/journal.pone.0269019 (PMC9159581; doi:10.1371/journal.pone.0269019)
Supplement: S3 Table — A. Multimodality imaging in 8 patients with discordant diagnoses. B. Multimodality imaging in 5 patients with additional findings. (DOCX) [file pone.0269019.s003.docx]

**S3 Table 3A Multimodality imaging in 8 patients with discordant diagnoses**

|  | **Post-transplant / histopathological diagnosis** | **Pre transplant/clinical diagnosis** | **Pre-transplant**  **echocardiogram** | **Pre-transplant coronary angiogram** | **Pre-transplant cardiac MRI** | **Pre-transplant EMB** |
| --- | --- | --- | --- | --- | --- | --- |
| Patient #1 | HCM | NISCM | Yes | Yes | **No** | **No** |
| Patient #2 | LDAC | NISCM | Yes | Yes | **No** | **No** |
| Patient #3 | ARVC | NISCM | Yes | Yes | Yes | **No** |
| Patient #4 | Cardiac sarcoidosis | ARVC | Yes | **No** | Yes | **No** |
| Patient #5 | Cardiac sarcoidosis | NISCM | Yes | Yes | Yes | **No** |
| Patient #6 | ISCM | NISCM | Yes | Yes | **No** | **No** |
| Patient #7 | LVNC | Myocarditis with cardiogenic shock, on ECMO | Yes | Yes | **No** | Yes |
| Patient #8 | Hypersensitivity myocarditis | Alcoholic CM | Yes | Yes | **No** | **No** |

ARVC: Arrhythmogenic right ventricular cardiomyopathy; CM: Cardiomyopathy, ECMO: extracorporeal membrane oxygenator; EMB: Endomyocardial biopsy; HCM: Hypertrophic cardiomyopathy; LDAC: left dominant arrhythmogenic cardiomyopathy; ISCM: Ischemic cardiomyopathy; LVNC: Left ventricular non-compaction cardiomyopathy; MRI: Magnetic Resonance Imaging; NISCM: non-ischemic cardiomyopathy

**S3 Table 3B Multimodality imaging in 5 patients with additional findings**

|  | **Main Histopathological diagnosis** | **Additional findings** | **Pre-transplant**  **echocardiography** | **Pre-transplant coronary angiogram** | **Pre-transplant cardiac MRI** | **Pre-transplant EMB** |
| --- | --- | --- | --- | --- | --- | --- |
| Patient A | NISCM | Coronary artery disease (50% LAD stenosis and old myocardial infarction scar in posterior wall) | Yes | Yes | No | No |
| Patient B | NISCM | Non-active myocarditis (small area) | Yes | Yes | No | No |
| Patient C | NISCM | Anomalous coronary artery | Yes | No | Yes | No |
| Patient D | NISCM | Coronary artery disease (50% stenosis of left main coronary artery) | Yes | Yes | No | No |
| Patient E | NISCM | Takayasu aortitis of the aorta | Yes | Yes | No | No |

CM: Cardiomyopathy; EMB: Endomyocardial biopsy; NISCM: non-ischemic cardiomyopathy
