# Supplementary figures and images for: The discrepancies between clinical and histopathological diagnoses of cardiomyopathies in patients with stage D heart failure undergoing heart transplantation
Source: PLoS One. 2022 Jun 1;17(6):e0269019. doi: 10.1371/journal.pone.0269019 (PMC9159581; doi:10.1371/journal.pone.0269019)

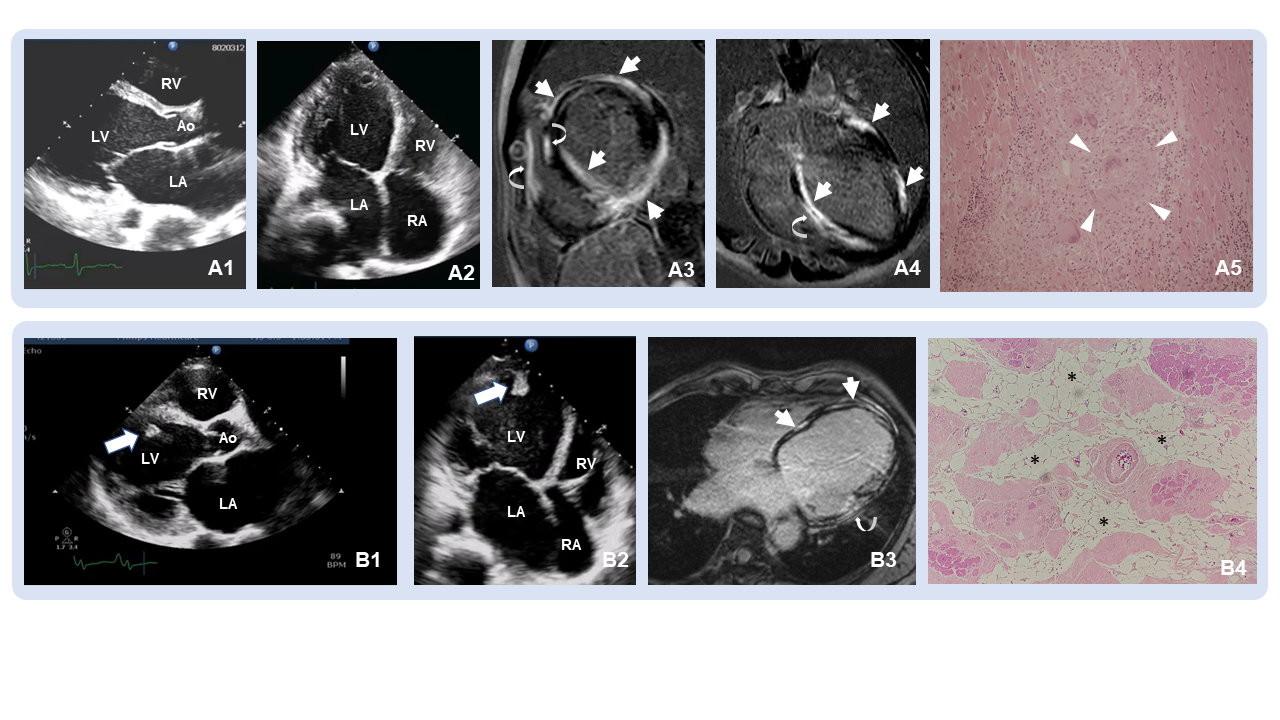

Supplement: S1 Fig — (TIF) [file pone.0269019.s004.tif]

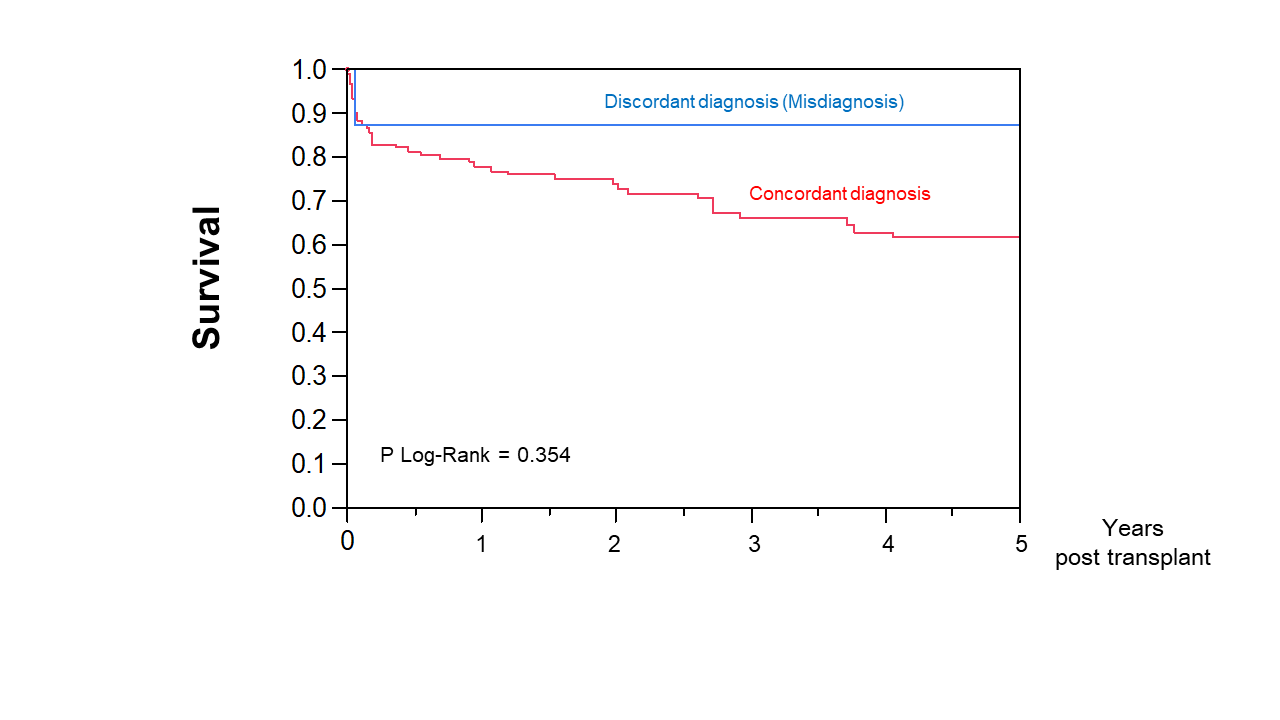

Supplement: S2 Fig — (TIF) [file pone.0269019.s005.tif]
