# Supplementary material for: The discrepancies between clinical and histopathological diagnoses of cardiomyopathies in patients with stage D heart failure undergoing heart transplantation
Source: PLoS One. 2022 Jun 1;17(6):e0269019. doi: 10.1371/journal.pone.0269019 (PMC9159581; doi:10.1371/journal.pone.0269019)
Supplement: S1 File — (DOCX) [file pone.0269019.s008.docx]

**Table S1:** Minimal data set for Table 1, 2, 3A, 3B, and figure 2

| **Patient number** | **Age (years)** | **Sex**  **(1= Male;**  **2 = Female)** | **Death**  **(1= yes; 0=no)** | **Time to event** | **Post-transplant diagnosis**  **(Pathological diagnosis)** | **Pre-transplant diagnosis** | **1= concordant 2= discordant** | **Additional diagnosis** | **Endomyocardial biopsy**  **(0=not done,**  **1 =done)** | **Cardiac MRI: (1=done, 0=not done)** | | **CAG: (1=done, 0=not done)** | | | **Bl. gr.** | **BW (kg)** | **Height (cm)** | | **BSA (m2)** |  |
| --- | --- | --- | --- | --- | --- | --- | --- | --- | --- | --- | --- | --- | --- | --- | --- | --- | --- | --- | --- | --- |
| 1 | 45 | 2 | 1 | 67 | 1 | 1 | 1 |  | 0 | 0 | | 1 | | | O | 48 | 153 | | 1.42828569 |  |
| 2 | 48 | 1 | 1 | 5771 | 1 | 2 | 2 |  | 0 | 0 | | 1 | | | O | 63 | 167 | | 1.7095321 |  |
| 3 | 26 | 1 | 1 | 3592 | 9 | 9 | 1 |  | 0 | 0 | | 1 | | | A | 42.9 | 160.5 | | 1.38297686 |  |
| 4 | 22 | 1 | 1 | 331 | 2 | 2 | 1 |  | 0 | 0 | | 1 | | | B | 90.5 | 174 | | 2.09145085 |  |
| 5 | 39 | 2 | 0 | 6664 | 2 | 2 | 1 |  | 0 | 0 | | 1 | | | O | 50 | 158 | | 1.48136574 |  |
| 6 | 20 | 1 | 0 | 6294 | 2 | 2 | 1 |  | 0 | 0 | | 1 | | | O | 99 | 170 | | 2.16217483 |  |
| 7 | 43 | 1 | 0 | 5833 | 2 | 2 | 1 |  | 0 | 0 | | 1 | | | B | 60 | 165 | | 1.6583124 |  |
| 8 | 26 | 1 | 1 | 953 | 3 | 3 | 1 |  | 0 | 0 | | 1 | | | O | 51.9 | 154.5 | | 1.49243928 |  |
| 9 | 55 | 1 | 1 | 1484 | 8 | 8 | 1 |  | 0 | 0 | | 1 | | | A | 61.5 | 165 | | 1.67891334 |  |
| 10 | 32 | 2 | 0 | 5002 | 4 | 4 | 1 |  | 0 | 0 | | 1 | | | B | 45.6 | 152 | | 1.38756381 |  |
| 11 | 53 | 2 | 1 | 720 | 1 | 1 | 1 |  | 0 | 0 | | 1 | | | B | 63 | 153 | | 1.63630682 |  |
| 12 | 49 | 1 | 1 | 1065 | 2 | 2 | 1 |  | 0 | 0 | | 0 | | | A | 51 | 165 | | 1.52888849 |  |
| 13 | 18 | 2 | 1 | 759 | 2 | 2 | 1 |  | 0 | 0 | | 0 | | | O | 48.2 | 160 | | 1.46363323 |  |
| 14 | 35 | 1 | 0 | 4586 | 7 | 4 | 2 |  | 0 | 1 | | 0 | | | B | 61 | 169 | | 1.69222076 |  |
| 15 | 52 | 1 | 1 | 992 | 2 | 2 | 1 |  | 0 | 0 | | 1 | | | O | 48 | 158 | | 1.45143607 |  |
| 16 | 14 | 2 | 0 | 4446 | 2 | 2 | 1 |  | 0 | 1 | | 0 | | | O | 36.5 | 158 | | 1.26567944 |  |
| 17 | 67 | 2 | 1 | 26 | 3 | 3 | 1 |  | 0 | 0 | | 1 | | | O | 55.4 | 143 | | 1.48344569 |  |
| 18 | 47 | 1 | 1 | 2314 | 2 | 2 | 1 |  | 0 | 0 | | 0 | | | B | 75 | 178 | | 1.92570333 |  |
| 19 | 37 | 1 | 1 | 27 | 1 | 1 | 1 |  | 0 | 0 | | 1 | | | B | 73 | 170 | | 1.85666966 |  |
| 20 | 46 | 2 | 1 | 2043 | 2 | 2 | 1 |  | 0 | 0 | | 1 | | | A | 47.5 | 161.5 | | 1.45976121 |  |
| 21 | 23 | 2 | 1 | 2 | 2 | 2 | 1 |  | 0 | 0 | | 1 | | | A | 45.6 | 165 | | 1.44568323 |  |
| 22 | 41 | 1 | 1 | 994 | 8 | 8 | 1 | Takayasu’s arteritis | 0 | 0 | | 0 | | | AB | 57 | 168 | | 1.63095064 |  |
| 23 | 59 | 2 | 1 | 7 | 8 | 8 | 1 |  | 0 | 0 | | 1 | | | O | 43 | 152 | | 1.34742553 |  |
| 24 | 57 | 1 | 1 | 165 | 1 | 1 | 1 |  | 0 | 1 | | 1 | | | O | 64.5 | 165 | | 1.71937489 |  |
| 25 | 44 | 1 | 1 | 2192 | 2 | 2 | 1 |  | 0 | 0 | | 1 | | | B | 76 | 172 | | 1.90554746 |  |
| 26 | 48 | 1 | 0 | 3576 | 1 | 1 | 1 |  | 0 | 0 | | 1 | | | A | 72 | 170 | | 1.84390889 |  |
| 27 | 31 | 1 | 1 | 18 | 2 | 2 | 1 |  | 0 | 0 | | 0 | | | B | 70 | 175 | | 1.84466197 |  |
| 28 | 57 | 2 | 1 | 20 | 2 | 2 | 1 | CAD | 0 | 0 | | 1 | | | B | 40 | 160 | | 1.33333333 |  |
| 29 | 48 | 1 | 0 | 3491 | 2 | 2 | 1 | Myocarditis in some area | 0 | 0 | | 1 | | | B+ | 58.5 | 172 | | 1.67182535 |  |
| 30 | 55 | 2 | 0 | 3478 | 2 | 2 | 1 |  | 0 | 0 | | 0 | | | B+ | 69 | 165 | | 1.77834192 |  |
| 31 | 45 | 2 | 1 | 132 | 6 | 6 | 1 |  | 0 | 0 | | 1 | | | O+ | 62 | 158 | | 1.64957907 |  |
| 32 | 27 | 1 | 0 | 3325 | 8 | 8 | 1 |  | 0 | 0 | | 0 | | | AB+ | 65 | 170 | | 1.751983 |  |
| 33 | 46 | 1 | 0 | 3269 | 1 | 1 | 1 |  | 0 | 1 | | 1 | | | A+ | 91 | 175 | | 2.10323824 |  |
| 34 | 24 | 2 | 0 | 3265 | 9 | 9 | 1 |  | 0 | 0 | | 0 | | | O+ | 71 | 153 | | 1.73709528 |  |
| 35 | 39 | 2 | 0 | 3207 | 1 | 1 | 1 |  | 0 | 0 | | 1 | | | B+ | 45 | 150 | | 1.36930639 |  |
| 36 | 27 | 2 | 0 | 3178 | 3 | 3 | 1 |  | 0 | 1 | | 0 | | | O+ | 36 | 149 | | 1.22065556 |  |
| 37 | 51 | 1 | 1 | 10 | 1 | 1 | 1 |  | 0 | 0 | | 1 | | | B+ | 66 | 163 | | 1.72867965 |  |
| 38 | 30 | 1 | 0 | 3074 | 2 | 2 | 1 |  | 0 | 0 | | 1 | | | A+ | 53 | 165 | | 1.55857841 |  |
| 39 | 29 | 1 | 1 | 1374 | 2 | 2 | 1 | Anomalous coronary artery | 0 | 1 | | 0 | | | O+ | 97 | 172 | | 2.15277599 |  |
| 40 | 39 | 1 | 0 | 2842 | 2 | 2 | 1 |  | 0 | 0 | | 1 | | | B+ | 58.4 | 175 | | 1.68490026 |  |
| 41 | 58 | 1 | 0 | 10 | 1 | 1 | 1 |  | 0 | 1 | | 1 | | | B+ | 55 | 165 | | 1.58771324 |  |
| 42 | 45 | 2 | 1 | 18 | 4 | 2 | 2 |  | 0 | 0 | | 1 | | | O+ | 44.6 | 146 | | 1.34490809 |  |
| 43 | 29 | 1 | 0 | 2726 | 9 | 9 | 1 |  | 0 | 1 | | 0 | | | B+ | 51 | 156 | | 1.48660687 |  |
| 44 | 48 | 2 | 0 | 2641 | 2 | 2 | 1 |  | 0 | 0 | | 0 | | | B+ | 41 | 161 | | 1.35410897 |  |
| 45 | 15 | 1 | 0 | 2605 | 4 | 4 | 1 |  | 0 | 0 | | 1 | | | O+ | 45 | 160 | | 1.41421356 |  |
| 46 | 53 | 1 | 0 | 2523 | 2 | 10-HSM | 2 |  | 0 | 0 | | 1 | | | O+ | 60.3 | 177 | | 1.72184494 |  |
| 47 | 17 | 2 | 0 | 2521 | 2 | 2 | 1 |  | 0 | 0 | | 0 | | | B+ | 41.8 | 158 | | 1.35445766 |  |
| 48 | 51 | 1 | 0 | 2505 | 1 | 1 | 1 |  | 0 | 1 | | 1 | | | O+ | 55 | 170 | | 1.61158997 |  |
| 49 | 55 | 1 | 1 | 69 | 1 | 1 | 1 |  | 0 | 0 | | 1 | | | O+ | 63.5 | 168 | | 1.72143351 |  |
| 50 | 23 | 2 | 0 | 2434 | 9 | 9 | 1 |  | 0 | 0 | | 1 | | | A+ | 32.5 | 155 | | 1.18292246 |  |
| 51 | 4 | 2 | 1 | 994 | 10 | 10 | 1 |  | 1 | 0 | | 0 | | | B+ | 14 | 100 | | 0.62360956 |  |
| 52 | 55 | 1 | 1 | 1356 | 2 | 2 | 1 |  | 0 | 0 | | 1 | | | O+ | 57 | 175 | | 1.66458203 |  |
| 53 | 56 | 1 | 0 | 2301 | 1 | 1 | 1 |  | 0 | 0 | | 1 | | | O+ | 60.4 | 170 | | 1.68885234 |  |
| 54 | 39 | 1 | 0 | 2276 | 9 | 9 | 1 |  | 0 | 1 | | 0 | | | O+ | 51 | 165 | | 1.52888849 |  |
| 55 | 47 | 1 | 1 | 562 | 1 | 1 | 1 |  | 0 | 0 | | 1 | | O+ | | 57 | | 172 | 1.65025251 | |
| 56 | 10 | 2 | 0 | 2177 | 3 | 3 | 1 |  | 1 | 1 | | | 1 | A+ | | 29.8 | | 140 | 1.07651702 | |
| 57 | 58 | 1 | 0 | 2157 | 10 | 10 | 1 |  | 1 | 1 | | | 1 | A+ | | 74.3 | | 174 | 1.89503738 | |
| 58 | 38 | 2 | 0 | 2152 | 3 | 3 | 1 |  | 0 | 0 | | | 1 | O+ | | 45.2 | | 167 | 1.44802548 | |
| 59 | 55 | 1 | 0 | 2441 | 1 | 1 | 1 |  | 0 | 0 | | | 1 | B+ | | 50 | | 162 | 1.5 | |
| 60 | 12 | 2 | 0 | 2128 | 9 | 9 | 1 |  | 0 | 0 | | | 0 | B+ | | 30.4 | | 143 | 1.09888833 | |
| 61 | 17 | 1 | 1 | 435 | 2 | 2 | 1 |  | 0 | 0 | | | 1 | B+ | | 70 | | 163 | 1.78029336 | |
| 62 | 51 | 1 | 0 | 2030 | 4 | 2 | 2 |  | 0 | 1 | | | 1 | A+ | | 44.5 | | 150 | 1.36167789 | |
| 63 | 31 | 2 | 0 | 2019 | 6 | 6 | 1 |  | 0 | 0 | | | 0 | O+ | | 41 | | 156 | 1.3329166 | |
| 64 | 45 | 1 | 0 | 1991 | 1 | 1 | 1 |  | 0 | 1 | | | 1 | A+ | | 70 | | 176 | 1.84992492 | |
| 65 | 27 | 1 | 1 | 12 | 2 | 2 | 1 |  | 0 | 0 | | | 0 | B+ | | 50 | | 168 | 1.52752523 | |
| 66 | 12 | 1 | 0 | 1916 | 2 | 2 | 1 |  | 0 | 0 | | | 1 | A+ | | 55 | | 166 | 1.59251722 | |
| 67 | 59 | 1 | 0 | 1882 | 1 | 1 | 1 |  | 0 | 1 | | | 1 | B+ | | 59.1 | | 167 | 1.65577273 | |
| 68 | 61 | 1 | 1 | 37 | 2 | 2 | 1 |  | 0 | 0 | | | 0 | A+ | | 72.1 | | 170 | 1.84518894 | |
| 69 | 50 | 1 | 0 | 1763 | 2 | 2 | 1 |  | 0 | 0 | | | 1 | O+ | | 63.1 | | 170 | 1.72618719 | |
| 70 | 16 | 2 | 0 | 1737 | 2 | 2 | 1 |  | 0 | 1 | | | 0 | A+ | | 69.8 | | 163 | 1.77774826 | |
| 71 | 36 | 1 | 0 | 1707 | 3 | 2 | 2 |  | 0 | 0 | 1 | | | O+ | | 60 | | 163 | 1.64823138 | |
| 72 | 21 | 1 | 0 | 1668 | 2 | 2 | 1 |  | 0 | 1 | 1 | | | B+ | | 48 | | 175 | 1.52752523 | |
| 73 | 53 | 1 | 1 | 21 | 1 | 1 | 1 |  | 0 | 0 | 0 | | | B+ | | 58 | | 189 | 1.74499284 | |
| 74 | 36 | 1 | 1 | 5 | 1 | 1 | 1 |  | 0 | 1 | 1 | | | A+ | | 68 | | 171 | 1.79722008 | |
| 75 | 47 | 2 | 0 | 1503 | 3 | 3 | 1 |  | 0 | 0 | 0 | | | O+ | | 65.5 | | 155 | 1.67932692 | |
| 76 | 55 | 1 | 1 | 389 | 1 | 1 | 1 |  | 0 | 0 | 1 | | | A+ | | 43.3 | | 160 | 1.38724347 | |
| 77 | 52 | 1 | 1 | 197 | 2 | 2 | 1 |  | 0 | 0 | 1 | | | O+ | | 47.8 | | 160 | 1.45754741 | |
| 78 | 16 | 2 | 1 | 254 | myxoma | myxoma | 1 |  | 1 | 0 | 0 | | | A+ | | 46.5 | | 147 | 1.37795138 | |
| 79 | 36 | 1 | 0 | 1380 | 2 | 2 | 1 |  | 0 | 1 | 1 | | | O+ | | 84.9 | | 172 | 2.01403409 | |
| 80 | 60 | 1 | 0 | 1319 | 2 | 2 | 1 |  | 0 | 1 | 1 | | | B+ | | 70.8 | | 158 | 1.76276298 | |
| 81 | 46 | 1 | 0 | 1285 | 2 | 2 | 1 | CAD | 0 | 0 | 1 | | | O+ | | 63.6 | | 167 | 1.71765344 | |
| 82 | 56 | 1 | 0 | 1252 | 1 | 1 | 1 |  | 0 | 1 | 1 | | | O+ | | 58.6 | | 172 | 1.67325365 | |
| 83 | 42 | 1 | 0 | 1232 | 4 | 4 | 1 |  | 1 | 1 | 1 | | | B+ | | 65.7 | | 171 | 1.76656446 | |
| 84 | 54 | 1 | 0 | 1231 | 2 | 2 | 1 |  | 0 | 0 | 0 | | | A+ | | 82 | | 160 | 1.90904281 | |
| 85 | 49 | 1 | 0 | 1225 | 2 | 2 | 1 |  | 0 | 0 | 1 | | | B+ | | 65.5 | | 170 | 1.75870849 | |
| 86 | 27 | 1 | 0 | 1203 | 2 | 2 | 1 |  | 0 | 0 | 1 | | | O+ | | 68.1 | | 170 | 1.79327447 | |
| 87 | 58 | 2 | 1 | 347 | 2 | 2 | 1 |  | 0 | 0 | 1 | | | O+ | | 43.2 | | 150 | 1.34164079 | |
| 88 | 51 | 1 | 0 | 1163 | 1 | 1 | 1 |  | 0 | 0 | 1 | | | A | | 51 | | 165 | 1.52888849 | |
| 89 | 29 | 1 | 0 | 1084 | 2 | 2 | 1 |  | 0 | 1 | 0 | | | AB | | 60 | | 170 | 1.68325082 | |
| 90 | 19 | 1 | 0 | 1078 | 2 | 2 | 1 |  | 0 | 0 | 0 | | | O+ | | 60 | | 166 | 1.66332999 | |
| 91 | 41 | 1 | 0 | 1065 | 2 | 2 | 1 |  | 0 | 1 | 0 | | | A+ | | 75 | | 175 | 1.90940654 | |
| 92 | 66 | 1 | 0 | 1049 | 1 | 1 | 1 |  | 0 | 0 | 1 | | | O+ | | 76.5 | | 169.5 | 1.89786064 | |
| 93 | 53 | 1 | 0 | 1043 | 1 | 1 | 1 |  | 0 | 1 | 1 | | | A+ | | 60 | | 170 | 1.68325082 | |
| 94 | 38 | 1 | 0 | 979 | 2 | 2 | 1 |  | 0 | 0 | 1 | | | B+ | | 47.5 | | 170 | 1.4976834 | |
| 95 | 51 | 1 | 0 | 959 | 2 | 2 | 1 |  | 0 | 0 | 1 | | | B+ | | 69.3 | | 169 | 1.8036768 | |
| 96 | 14 | 1 | 0 | 907 | 4 | 4 | 1 |  | 0 | 0 | 0 | | | A+ | | 48.9 | | 163 | 1.48797961 | |
| 97 | 19 | 1 | 1 | 733 | 8 | 8 | 1 |  | 0 | 1 | 0 | | | O+ | | 53.6 | | 166 | 1.57211817 | |
| 98 | 11 | 1 | 1 | 63 | 2 | 2 | 1 |  | 0 | 1 | 1 | | | A+ | | 27.4 | | 149 | 1.06492045 | |
| 99 | 53 | 1 | 0 | 797 | 1 | 1 | 1 |  | 0 | 1 | 1 | | | B+ | | 50.7 | | 163 | 1.51511826 | |
| 100 | 53 | 1 | 0 | 772 | 2 | 2 | 1 |  | 0 | 0 | 1 | | | O+ | | 61.7 | | 169 | 1.70190253 | |
| 101 | 47 | 1 | 0 | 747 | 2 | 2 | 1 |  | 0 | 0 | 1 | | | B+ | | 56 | | 170 | 1.62617479 | |
| 102 | 30 | 1 | 0 | 719 | 7 | 2 | 2 |  | 0 | 1 | 1 | | | O+ | | 52.4 | | 168 | 1.56375616 | |
| 103 | 50 | 1 | 0 | 694 | 2 | 2 | 1 |  | 0 | 0 | 1 | | | B+ | | 66 | | 166 | 1.74451521 | |
| 104 | 34 | 1 | 0 | 694 | 1 | 1 | 1 |  | 0 | 0 | 1 | | | A+ | | 73 | | 175 | 1.88377576 | |
| 105 | 67 | 2 | 1 | 4 | 8 | 8 | 1 |  | 0 | 0 | 1 | | | B+ | | 55 | | 161 | 1.56835016 | |
| 106 | 60 | 1 | 1 | 14 | 1 | 1 | 1 |  | 0 | 0 | 1 | | | O+ | | 55 | | 160 | 1.56347192 | |
| 107 | 52 | 1 | 0 | 625 | 2 | 2 | 1 |  | 0 | 0 | 1 | | | B+ | | 79 | | 175 | 1.95966267 | |
| 108 | 50 | 1 | 0 | 624 | 2 | 2 | 1 |  | 0 | 0 | 1 | | | B+ | | 63.5 | | 164 | 1.7008168 | |
| 109 | 52 | 1 | 0 | 612 | 2 | 2 | 1 |  | 0 | 0 | 1 | | | AB+ | | 46.5 | | 162 | 1.44654761 | |
| 110 | 46 | 2 | 1 | 61 | 1 | 1 | 1 |  | 0 | 0 | 1 | | | O+ | | 65 | | 160 | 1.69967317 | |
| 111 | 46 | 1 | 0 | 573 | 1 | 1 | 1 |  | 0 | 0 | 1 | | | A+ | | 64.8 | | 167 | 1.73378199 | |
| 112 | 54 | 1 | 0 | 473 | 2 | 2 | 1 |  | 0 | 1 | 1 | | | AB+ | | 54 | | 165 | 1.57321327 | |
| 113 | 62 | 2 | 0 | 447 | 2 | 2 | 1 |  | 0 | 1 | 1 | | | A+ | | 53.3 | | 160 | 1.53911952 | |
| 114 | 44 | 2 | 0 | 441 | 2 | 2 | 1 |  | 0 | 0 | 0 | | | B+ | | 41 | | 162 | 1.35830777 | |
| 115 | 50 | 1 | 0 | 414 | 1 | 1 | 1 |  | 0 | 0 | 1 | | | B+ | | 50 | | 165 | 1.51382518 | |
| 116 | 47 | 1 | 0 | 398 | 5 | 10-myocarditis | 2 |  | 1 | 0 | 1 | | | O+ | | 66.1 | | 168 | 1.75632191 | |
| 117 | 37 | 1 | 1 | 55 | 2 | 2 | 1 |  | 0 | 0 | 1 | | | AB+ | | 52 | | 167 | 1.55313303 | |
| 118 | 56 | 2 | 0 | 383 | 2 | 2 | 1 |  | 0 | 0 | 1 | | | O+ | | 53 | | 162 | 1.54434452 | |
| 119 | 59 | 2 | 0 | 378 | 2 | 2 | 1 |  | 0 | 0 | 1 | | | B+ | | 47.8 | | 155 | 1.43459247 | |
| 120 | 53 | 1 | 0 | 360 | 8 | 8 | 1 |  | 0 | 0 | 1 | | | O+ | | 45.4 | | 160 | 1.42048505 | |
| 121 | 60 | 1 | 1 | 21 | 8 | 8 | 1 |  | 0 | 0 | 1 | | | A+ | | 54 | | 163 | 1.56364958 | |
| 122 | 39 | 1 | 0 | 268 | 2 | 2 | 1 |  | 0 | 0 | 1 | | | B+ | | 69 | | 175 | 1.83143841 | |
| 123 | 23 | 2 | 1 | 13 | 3 | 3 | 1 |  | 0 | 0 | 1 | | | B+ | | 51.8 | | 162 | 1.52676128 | |
| 124 | 36 | 1 | 0 | 230 | 2 | 2 | 1 |  | 0 | 0 | 1 | | | O+ | | 70 | | 170 | 1.81811869 | |
| 125 | 5 | 1 | 0 | 217 | 10 | 10 | 1 |  | 0 | 0 | 0 | | | A+ | | 12.6 | | 105 | 0.60621778 | |
| 126 | 65 | 1 | 0 | 185 | 1 | 1 | 1 |  | 0 | 1 | 1 | | | B+ | | 66 | | 164 | 1.73397424 | |
| 127 | 34 | 2 | 0 | 35 | 2 | 2 | 1 |  | 0 | 1 | 0 | | | AB+ | | 49 | | 165 | 1.49861047 | |
